# Marginalized Gromov Flow Particle Filter-Based Track-Before-Detect Algorithm for Marine Targets

**DOI:** 10.3390/s26206624

**Published:** 2026-10-19

**Authors:** Fan Zhang, Chang Liu

**Affiliations:** 1 Aerospace Information Research Institute, Chinese Academy of Sciences, Beijing 100190, China; messier72@protonmail.com; 2 School of Electronic, Electrical and Communication Engineering, University of Chinese Academy of Sciences, Beijing 100049, China

**Keywords:** target tracking, track-before-detect, marginalized particle filter, particle flow particle filter

## Abstract

Marine target tracking suffers from many difficulties such as weak echoes and complex background environments. In this paper, a marginalized Gromov particle flow particle filter-based track-before-detect (MPFPF-TBD) algorithm is proposed. Firstly, the invertible Gromov particle flow is utilized to incorporate the observation information, aiming to optimize proposal distribution. Secondly, the target state space is divided using a marginalization strategy, so the particle flow and extended Kalman methods can be utilized to calculate the nonlinear measurement-related state component and the conditionally linear state component, respectively. Finally, the K-distribution is applied to design the marine clutter model, and the track-before-detect method is utilized for energy accumulation. This algorithm is evaluated using simulated targets in simulated and measured sea clutter. The results indicate that, compared with the particle method, the LEDH-based particle flow method and the SFA-PF-TBD method, this algorithm exhibits advantages in estimation performance, computational efficiency and robustness.

## 1. Introduction

Tracking weak targets is a challenging problem in radar target tracking. When the target echo is submerged in strong background clutter and noise, detection may produce a large number of false alarms, leading to a combinatorial explosion in the data association stage. Various techniques have been investigated to improve weak target detection and tracking performance, such as adaptive clutter suppression [1], long-time coherent integration [2], and multi-sensor fusion [3]. Among these methods, track-before-detect (TBD) is an effective target tracking approach compared to the traditional detect-before-track (DBT) method. TBD methods can be broadly divided into two categories: batch processing methods, such as dynamic programming [4] and Hough transform [5], and recursive methods, such as particle-based approaches [6,7,8] and random finite set methods [9,10]. Among these, particle-based methods have attracted some attention due to their relatively low computational and memory costs.

Methods based on particle filters are generally affected by the particle degeneracy problem, where after a few iterations, the weights concentrate on a small number of particles. The root cause of particle degeneracy lies in the pointwise product of probabilities during Bayesian recursion. Therefore, using an optimal importance function can effectively alleviate this problem. Since the optimal importance function is often difficult to sample or lacks an analytical solution, approximate density functions are commonly used, such as the extended particle filter [11] and the unscented particle filter [12]. These methods perform well when the prediction and posterior distributions have specific forms. Another approach is to approximate the predictive likelihood function using current observation information, such as the auxiliary particle filter [13]. However, it can be shown that when background noise is significant, this method does not improve estimation performance [14]. This limitation motivates the need for alternative update mechanisms that avoid weight computation altogether.

To address the above issues, Daum [15] proposed the theory of particle flow filtering, which utilizes homotopy to gradually transition particles from the prior probability to the posterior distribution, thereby avoiding the pointwise product of particle weights and eliminating the need to compute weights. This theory assumes that particle migration follows an Itô stochastic process, obtains the flow function by solving a set of differential equations, and gradually integrates over pseudo-time steps to obtain the posterior distribution. Depending on the assumptions, this differential equation has multiple solutions, such as EDH [16], Geodesic [17], and Gromov [18], all of which offer advantages over PF in terms of estimation performance. The application of particle flow filtering theory can be categorized into two approaches. One approach involves directly using particle flow filters to process measurement data [19,20]. In this method, particle flow is applied during the update stage of Bayesian filtering. The other approach utilizes the output of particle flow as a proposal distribution, known as the Particle Flow Particle Filter (PFPF) [21]. PFPF executes a particle flow migration to obtain a particle set and forms a high-quality proposal distribution for standard particle filtering steps, including importance sampling and resampling. In [22], a Gromov stochastic flow incorporating a non-zero diffusion term is employed to construct the proposal distribution, and it is proven that the introduction of resampling helps enhance tracking performance. In [23], the Particle Flow Gaussian Particle Filter (PFGPF) leverages reversible particle flow to migrate particles within the pseudo-time domain, generating a Gaussian proposal distribution that closely approximates the posterior distribution, thereby mitigating particle degeneracy issues in high-dimensional state spaces. More recently, PFPF has been integrated with Gaussian Mixture Models (GMMs) and applied within the Belief Propagation (BP) framework [24]. Compared to classical methods, these particle flow-based TBD approaches offer better estimation performance but suffer from lower computational efficiency, so they typically use only a small number of particles to fit the target distribution. In weak target tracking, however, a sufficient number of particles is essential to respond to target birth and death quickly [25]. Balancing estimation performance and computational efficiency in particle flow-based TBD methods is therefore a topic of research value.

Marginalized particle filtering is an efficient hybrid inference framework that combines analytical recursion with stochastic sampling. Its core idea leverages the conditional independence structure of the target state space, using a structured decomposition to transform a high-dimensional filtering problem into a process of “low-dimensional sampling and high-dimensional analytical updating.” This approach significantly reduces computational complexity while maintaining estimation accuracy. The method has been applied to tasks such as SLAM [26], autonomous driving [27], extended objects [28], and underwater target tracking [29]. In [30], its performance within a deterministic particle flow with a zero diffusion term has been investigated. However, to the best of our knowledge, this strategy has not yet been extended to stochastic flows that include non-zero diffusion terms, nor has its effect in track-before-detect applications been explored.

This paper proposes a marginalized Gromov flow particle filter-based track-before-detect algorithm (MPFPF-TBD). The algorithm first uses the Gromov flow method to guide particle migration, alleviating the particle degeneracy problem. Additionally, it employs marginalization to partition the target state space, separately estimating target position, velocity, and acceleration. Finally, the K-distribution is used to model the sea clutter background, and the TBD framework is applied to achieve inter-frame energy accumulation for weak targets. The proposed method is simulated in sea surface target tracking scenarios with varying clutter intensities and state-space dimensions and tested using measured sea clutter data with synthetically injected target echoes. The results demonstrate the algorithm’s effective performance.

## 2. System Model

### 2.1. Motion Model

Suppose at time *k*, a single target exists in the region of interest detected by a marine surveillance radar. In the surveillance area, the evolution of the target state is described with the constant acceleration (CA) motion model, whose state is fully characterized by $x_{k}$.

(1) $$x_{k}=F_{k}x_{k-1}+v_{k}$$

For the CA model, the state vector $x_{k}={\left[x_{k},{\dot{x}}_{k},{\ddot{x}}_{k},y_{k},{\dot{y}}_{k},{\ddot{y}}_{k}\right]}^{T}$ includes position, velocity, and acceleration in a two-dimensional plane. Here, $v_{k}$ is a zero-mean white Gaussian process noise, and $F_{k}$ is the state transition matrix.

Let the set $E_{k}\in \left\{e_{k},{\bar{e}}_{k}\right\}$ denote the existence of the target at time *k*. The state transition is modeled as a binary Markov process, and the probabilities of target birth and death are defined as follows:

(2) $$\left\{\begin{array}{c} P_{b}=P\left\{E_{k}=e_{k}|\;\;E_{k-1}={\bar{e}}_{k-1}\right\}\\ P_{d}=P\left\{E_{k}={\bar{e}}_{k}|\;\;E_{k-1}=e_{k-1}\right\} \end{array}\right.$$

The state transition probability is given by

(3) $$\begin{array}{c} \prod_{e}= \end{array}\left(\begin{array}{cc} 1-P_{b} & P_{b}\\ P_{d} & 1-P_{d} \end{array}\right)$$

### 2.2. Measurement Model

At time *k*, the radar scans the target to obtain two-dimensional measurements in range and azimuth. A single scan includes $N_{r}*N_{b}$ resolution cells. The set of measurement amplitudes is defined as $z_{k}=\left\{z_{k}^{\left(i,j\right)},i=1,2,\cdots ,N_{r},j=1,2,\cdots ,N_{b}\right\}$. In the super-measurement model commonly used in TBD, the measured amplitude in the resolution cell $\left(i,j\right)$ corresponds to the complex envelope resulting from the superposition of the target echo and clutter. The signal amplitude $z_{k}^{\left(i,j\right)}$ of this envelope can be expressed as follows:

(4) $$z_{k}^{\left(i,j\right)}=\left|z_{c,k}^{\left(i,j\right)}\right|=\left\{\begin{array}{cc} \left|A_{k}h_{A}^{\left(i,j\right)}\left(x_{k}\right)+v_{k}^{\left(i,j\right)}\right|, & E_{k}=e_{k}\\ \left|v_{k}^{\left(i,j\right)}\right|, & E_{k}={\bar{e}}_{k} \end{array}\right.$$

where $z_{c,k}^{\left(i,j\right)}$ represents the complex amplitude of the measurement cell $\left(i,j\right)$, $A_{k}\left(\cdot \right)$ is the complex amplitude function of the target, and $h_{A}^{\left(i,j\right)}\left(\cdot \right)$ denotes the complex amplitude spread function of the target within the cell $\left(i,j\right)$. The target’s Radar Cross Section (RCS) is modeled using the Swerling 0 model, $A_{k}=\bar{A}e^{j\theta }$, where $\bar{A}$ is the echo amplitude and $\theta \sim U\left(0,2\pi \right)$ is the phase. $v_{k}^{\left(i,j\right)}$ is the observation noise.

Next, the measurement likelihood function is discussed under two scenarios. For the scenario with no target, the radar receives only sea-surface clutter, whose statistical characteristics are influenced by factors such as radar wavelength, sea state, incidence angle, and weather conditions. Currently, the K-distribution model is frequently used to characterize the amplitude statistics, defined as follows:

(5) $$p\left(z_{k}|\;E_{k}={\bar{e}}_{k}\right)=\frac{4{\left(z_{k}\right)}^{\alpha }}{{\sqrt{2\beta }}^{\alpha +1}\Gamma \left(\alpha \right)}K_{\alpha -1}\left(\frac{2z_{k}}{\sqrt{2\beta }}\right)$$

where $\Gamma \left(\cdot \right)$ is the Gamma function, $K_{\alpha -1}\left(\cdot \right)$ is the modified Bessel function of the second kind, and α, β are the shape and scale parameters of the K-distribution, respectively. By adjusting the shape and scale parameters, the pdf of sea clutter amplitudes under various conditions can be accurately modeled.

For scenarios in which a target exists, the complex amplitude spread function $h_{A}^{\left(i,j\right)}\left(\cdot \right)$ in (4) is defined as follows:

(6) $$h_{A}^{\left(i,j\right)}\left(x_{k}\right)\approx \frac{\Delta_{r}\Delta_{b}A_{k}}{2\pi \delta_{k}^{2}}\exp\left\{-\frac{{\left(i\Delta_{r}-r\left(x_{k}\right)\right)}^{2}}{2\Sigma_{r}^{2}}-\frac{{\left(j\Delta_{b}-b\left(x_{k}\right)\right)}^{2}}{2\Sigma_{b}^{2}}\right\}$$

where $\Delta_{r},\Delta_{b}$ represent the dimensions of the range and azimuth resolution cells, respectively; $\Sigma_{r}^{2},\Sigma_{b}^{2}$ describe the radar’s range and azimuth resolution, respectively; $\delta_{k}^{2}$ denotes the intensity-modified ambiguity function; and $\left\{r\left(x_{k}\right),b\left(x_{k}\right)\right\}$ are the range-azimuth measurement coordinates of the target, denoted as

(7) $$\left[\begin{array}{c} r\left(x_{k}\right)\\ b\left(x_{k}\right) \end{array}\right]=\left[\begin{array}{c} \sqrt{x_{k}^{2}+y_{k}^{2}}\\ \arctan\left(\frac{y_{k}}{x_{k}}\right) \end{array}\right]$$

When a target exists in the measurement region, the likelihood function is given by the following [31]:

(8) $$p\left(z_{k}|\;x_{k},E_{k}=e_{k}\right)=\frac{z_{k}}{\beta^{\alpha }\Gamma \left(\alpha \right)}\int_{0}^{\infty }\eta^{\alpha -2}I_{0}\left(\frac{A_{k}z_{k}}{\eta }\right)\exp\left(-\frac{z_{k}^{2}+A_{k}^{2}}{2\eta }-\frac{\eta }{\beta }\right)d\eta$$

where $I_{0}\left(\cdot \right)$ is the modified Bessel function of the second kind. For the integral in (8), piecewise interpolation can be used to approximate the solution.

Using the measurement model established by (4)–(8), the likelihood ratio function of a resolution cell is defined as follows:

(9) $$l\left(z_{k}^{\left(i,j\right)}|\;x_{k},E_{k}\right)=\left\{\begin{array}{c} \frac{p\left(z_{k}^{\left(i,j\right)}|\;x_{k},E_{k}=e_{k}\right)}{p\left(z_{k}^{\left(i,j\right)}|\;E_{k}={\bar{e}}_{k}\right)}E_{k}=e_{k}\\ 1\;\;\;\;\;\;\;E_{k}={\bar{e}}_{k} \end{array}\right.$$

Under the adopted likelihood model, the measurement noise is modeled as statistically independent between resolution cells, and the measurement set $z_{k}$ is conditionally independent given the target state and existence variable. The influence of the target measurement signal is spatially limited. Consequently, the measurement likelihood function can be approximated by a local likelihood. Let the neighborhood ranges affected by the target in the range and azimuth dimensions be $C_{i}(x_{k})$ and $C_{j}(x_{k})$, respectively. Then, the local measurement likelihood ratio function for the region is defined as

(10) $$l\left(z_{k}|x_{k},E_{k}\right)=\underset{i\in c_{i}\left(x_{k}\right)}{\prod }\underset{j\in c_{j}\left(x_{k}\right)}{\prod }l\left(z_{k}^{\left(i,j\right)}|x_{k},A_{k},E_{k}\right)$$

## 3. Marginalized Gromov Flow Particle Filter-Based TBD

### 3.1. Particle Flow Filtering

The particle flow is defined by a logarithmic homotopy transformation similar to the following Bayesian update equation [32]:

(11) $$\ln p\left(x_{k},\lambda \right)=\ln g\left(x_{k}\right)+\lambda \ln h\left(x_{k}\right)-\ln K\left(\lambda \right)$$

where $p\left(x_{k},\lambda \right)$ is the posterior probability density function, $g\left(x_{k}\right)$ is the prior probability density, $h\left(x_{k}\right)$ is the likelihood function, $\lambda \in \left[0,1\right]$ is the pseudo-time step, and $K\left(\lambda \right)$ is the normalization constant, expressed as follows:

(12) $$K\left(\lambda \right)=\int g\left(x_{k}\right)h{\left(x_{k}\right)}^{\lambda }\;dx_{k}$$

In (11), a transformation process from the prior probability density to the posterior probability density is defined. As λ increases from 0 to 1, the particles evolve from the prior distribution to the posterior distribution. This transformation is modeled as an Ito stochastic process with respect to the pseudo-time step, expressed as follows:

(13) $$dx_{k}=f\left(x_{k},\lambda \right)d\lambda +\sigma \left(x_{k},\lambda \right)dw$$

where $f\left(x_{k},\lambda \right)$ is the drift function, and $\sigma \left(x_{k},\lambda \right)$ is the diffusion term. Applying the Fokker–Planck equation to this expression gives the following:

(14) $$\frac{\partial p\left(x_{k},\lambda \right)}{\partial \lambda }=-\mathrm{div}\left(f\left(x_{k},\lambda \right)p\left(x_{k},\lambda \right)\right)+\frac{1}{2}\mathrm{div}\left[Q\left(x_{k},\lambda \right)\frac{\partial p\left(x_{k},\lambda \right)}{\partial x_{k}}\right]$$

where $Q\left(x_{k},\lambda \right)$ is the diffusion covariance matrix, satisfying $Q\left(x_{k},\lambda \right)=\sigma \left(x_{k},\lambda \right)\sigma {\left(x_{k},\lambda \right)}^{T}$, and div denotes the divergence operator. Depending on the assumptions, the solution to (14) is not unique. The solution employed here is referred to as the Gromov flow [18], whose drift function is given by the following:

(15) $$f\left(x_{k},\lambda \right)=-{\left[\frac{\partial^{2}\ln p\left(x_{k},\lambda \right)}{\partial x_{k}^{2}}\right]}^{-1}{\left(\frac{\partial \ln h\left(x_{k}\right)}{\partial x_{k}}\right)}^{T}$$

where the diffusion covariance matrix solution is given by

(16) $$Q\left(x_{k},\lambda \right)=-{\left[\frac{\partial^{2}\ln p\left(x_{k},\lambda \right)}{\partial x_{k}^{2}}\right]}^{-1}\frac{\partial^{2}\ln h\left(x_{k}\right)}{\partial x_{k}^{2}}{\left[\frac{\partial^{2}\ln p\left(x_{k},\lambda \right)}{\partial x_{k}^{2}}\right]}^{-1}$$

To enable particles to “flow” from the prior distribution to the posterior distribution, the pseudo-time term is integrated over the interval during the Bayesian update stage using (15) and (16). This integration is carried out via interpolation, expressed as follows:

(17) $$\eta_{\lambda_{j}}=\eta_{\lambda_{j-1}}+\epsilon_{j}f\left(x_{k},\lambda_{j}\right)+\sqrt{\epsilon_{j}}v\;\left(x_{k},\lambda_{j}\right)$$

where $\lambda_{j}\in \left[0,1\right]$ is the pseudo-time step, and $\eta_{\lambda_{j}}$ represents a single particle in the pseudo-time integration. Specifically, $\eta_{0}$ and $\eta_{1}$ denote a single particle in the prior and posterior distributions, respectively. $\epsilon_{j}$ is the pseudo-time step size, defined as $\epsilon_{j}=\lambda_{j}-\lambda_{j-1}\;$, $v\left(x_{k},\lambda_{j}\right)\sim N\left(0,Q\left(x_{k},\lambda_{j}\right)\right)$. The particle flow particle filter uses the output of the particle flow guided by (17) as the input for weight calculation, essentially improving the proposal distribution [22].

### 3.2. Marginalized Gromov Flow Particle Filtering

The computational burden of Gromov particle flow increases rapidly with the state dimension because the flow parameters require repeated matrix operations. Marginalization is introduced here to reduce the dimension on which stochastic sampling and Gromov flow integration are performed, while retaining the full physical target state.

Let the target state be partitioned into a measurement-related nonlinear component $x_{k,a}$ and a conditionally linear component $x_{k,b}$. For the marine radar model considered here, the former contains the position variables, whereas the latter contains the higher-order kinematic variables. Denoting the measurement-related nonlinear state trajectory by $x_{0:k,a}$, the joint posterior can be written exactly by the probability chain rule as

(18) $$p\left(x_{0:k,a},x_{k,b}|\;z_{1:k}\right)=p\left(x_{k,b}|\;x_{0:k,a},z_{1:k}\right)p\left(x_{0:k,a}|\;z_{1:k}\right)$$

The marginalized formulation relies on properties of the adopted state-space model. In (6) and (7), the current measurement likelihood depends directly on $x_{k,a}$ only. This means the corresponding conditional-independence relation is

(19) $$p\left(z_{k}|\;x_{0:k,a},x_{k,b},z_{1:k-1}\right)=p\left(z_{k}|\;x_{k,a}\right)$$

With this property, the role of marginalization in the Bayesian update can be stated directly. Bayes’ rule gives the joint posterior

(20) $$p\left(x_{0:k,a},x_{k,b}|\;z_{1:k}\right)=\frac{p\left(z_{k}|\;x_{k,a}\right)p\left(x_{0:k,a},x_{k,b}|\;z_{1:k-1}\right)}{p\left(z_{k}|\;z_{1:k-1}\right)}$$

Integrating (20) over the conditionally linear component gives the marginalized recursion for the nonlinear component

(21) $$p\left(x_{0:k,a}|\;z_{1:k}\right)=\int p\left(x_{0:k,a},x_{k,b}|\;z_{1:k}\right)dx_{k,b}=\frac{p\left(z_{k}|\;x_{k,a}\right)p\left(x_{0:k,a}|\;z_{1:k-1}\right)}{p\left(z_{k}|\;z_{1:k-1}\right)}$$

This means the particle-flow particle filter samples and transports particles only in the nonlinear measurement-related subspace. The conditionally linear component is retained through its conditional density, represented with

(22) $$p\left(x_{k,b}|\;x_{0:k,a},z_{1:k}\right)=p\left(x_{k,b}|\;x_{0:k,a},z_{1:k-1}\right)$$

Thus, the current measurement affects $x_{k,b}$ indirectly through the updated distribution of $x_{0:k,a}$ and through the dynamic coupling between the two state partitions. The marginalization therefore reduces the stochastic sampling dimension without discarding the posterior information carried by the conditionally linear state component.

**Figure 1:**
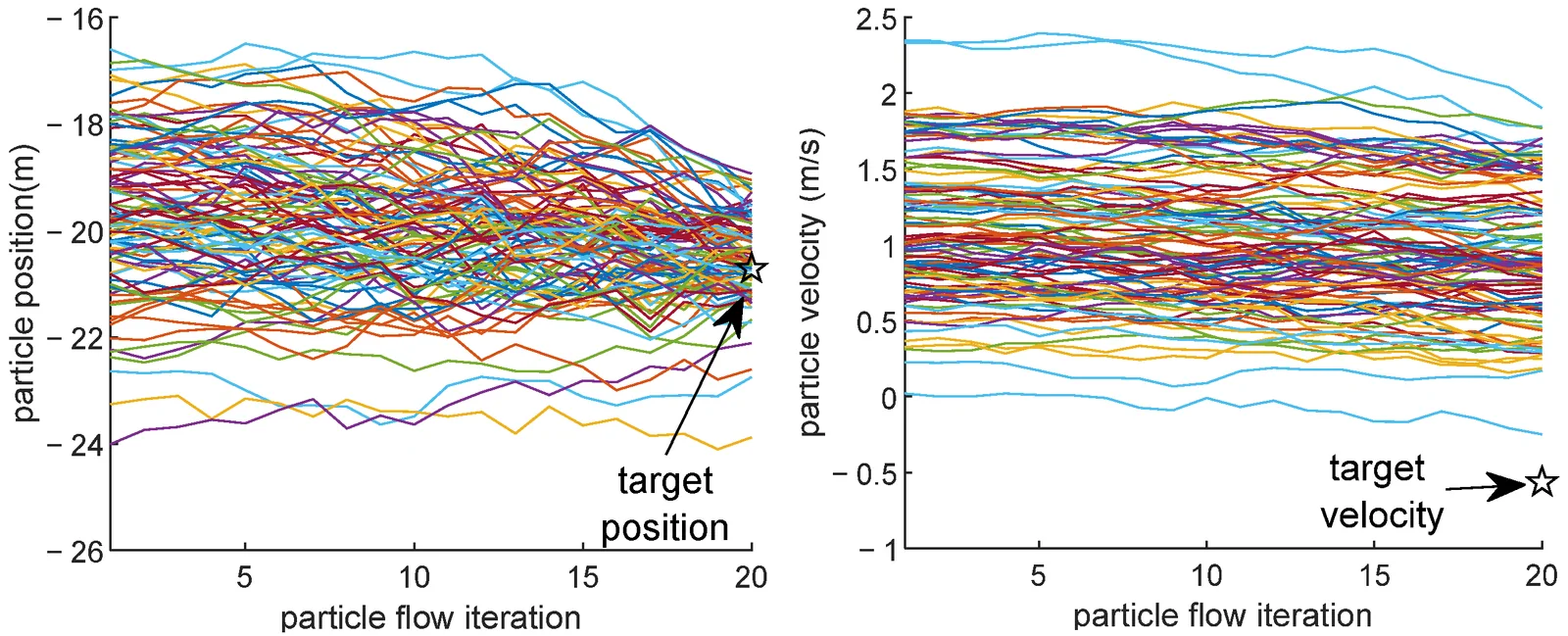
Flow iteration process of target position and velocity, the different color lines indicate different particle evolution trajectories.

In Figure 1, the process of particles forming a proposal distribution is represented by a cluster of polylines, and the true target state is marked with a star. It can be seen that integrating the Gromov flow function directly changes the sampled position components, whereas velocity is handled through the conditional state update rather than the particle flow integration.

Based on the above reasons, a novel marginalized Gromov flow particle filter algorithm is proposed here, and the system equations are defined as follows [33]:

(23) $$\begin{array}{l} x_{k,a}=F_{aa}x_{k-1,a}+F_{ab}x_{k-1,b}+v_{k-1,a}\\ x_{k,b}=F_{ba}x_{k-1,a}+F_{bb}x_{k-1,b}+v_{k-1,b}\\ z_{k}\;=h\left(x_{k,a}\right)+w_{k} \end{array}$$

where $x_{k}={\left[{x_{k,a}}^{T},{x_{k,b}}^{T}\right]}^{T}$, $x_{k,a}$ is the nonlinear measurement-related component at time *k*, and $x_{k,b}$ is the conditional linear component at time *k*, which can be computed using an extended Kalman Filter. $v_{k-1,a}$ and $v_{k-1,b}$ denote the process noises for the two corresponding components. $w_{k}$ denotes the measurement noise. $F=\left[\left[F_{aa},F_{ab}];[F_{ba},F_{bb}\right]\right]$ is the block-partitioned state transition matrix, where its subscripts correspond to the partitions associated with $x_{k,a}$ and $x_{k,b}$. For the CA model, $x_{k,a}={\left[x_{k},y_{k}\right]}^{T}$, $x_{k,b}={\left[{\dot{x}}_{k},{\ddot{x}}_{k},{\dot{y}}_{k},{\ddot{y}}_{k}\right]}^{T}$, and $F_{aa},F_{ab},F_{ba},F_{bb}$ are denoted as follows:

(24) $$F_{aa}=\left[\begin{array}{cc} 1 & 0\\ 0 & 1 \end{array}\right]\;\;F_{ab}=\left[\begin{array}{llll} T & \frac{T^{2}}{2} & 0 & 0\\ 0 & 0 & T & \frac{T^{2}}{2} \end{array}\right]\;F_{ba}=\left[\begin{array}{ll} 0 & 0\\ 0 & 0\\ 0 & 0\\ 0 & 0 \end{array}\right]\;\;\;\;\;F_{bb}=\left[\begin{array}{cccc} 1 & T & 0 & 0\\ 0 & 1 & 0 & 0\\ 0 & 0 & 1 & T\\ 0 & 0 & 0 & 1 \end{array}\right]$$

Physically, the current target likelihood depends directly on the relative position between the radar and the target, while higher-order motion states such as velocity and acceleration influence the measurement only indirectly through the dynamic evolution of position. For the conditionally linear component, the conditional prior is represented by a Gaussian distribution:

(25) $$p\left(x_{k,b}|x_{k-1,a},x_{k-1,b}\right)=N\left({\bar{x}}_{k|k-1,b},{\bar{P}}_{k|k-1,bb}\right)$$

where

(26) $${\bar{x}}_{k|k-1,b}=F_{ba}x_{k-1,a}+F_{bb}x_{k-1,b}$$

(27) $$\begin{array}{ll} {\bar{P}}_{k|k-1,bb} & =F_{ba}P_{k-1|k-1,aa}F_{ba}^{T}+F_{bb}P_{k-1|k-1,ba}F_{ba}^{T}\\ & +F_{ba}P_{k-1|k-1,ab}F_{bb}^{T}+F_{bb}P_{k-1|k-1,bb}F_{bb}^{T}+Q_{k-1,bb} \end{array}$$

where $P_{k-1|k-1}=\left[\left[P_{k-1|k-1,aa},P_{k-1|k-1,ab}];[P_{k-1|k-1,ba},P_{k-1|k-1,bb}\right]\right]$, $P_{k-1|k-1,aa}$, $P_{k-1|k-1,ab}$, $P_{k-1|k-1,ba}$, and $P_{k-1|k-1,bb}$ denote the covariance matrix of the nonlinear component, the cross-covariance matrices between the linear and nonlinear components, and the covariance matrix of the linear component, respectively. $Q_{k-1,bb}$ represents the process noise covariance matrix of the conditionally linear component. For the system model given in (23), the conditionally linear component is updated in (28)–(31):

(28) $$x_{k|k,b}=x_{k|k-1,b}+L_{k}\left(x_{k|k,a}-x_{k|k-1,a}\right)$$

(29) $$P_{k|k,bb}=P_{k|k-1,bb}+L_{k}\left(P_{k|k,aa}-P_{k|k-1,aa}\right)L_{k}^{T}$$

where

(30) $$L_{k}=P_{k|k-1,ba}{\left(P_{k|k-1,aa}\right)}^{-1}$$

(31) $$\begin{array}{ll} P_{k|k-1,ba} & =F_{ba}P_{k-1|k-1,aa}F_{aa}^{T}+F_{bb}P_{k-1|k-1,ba}F_{aa}^{T}\\ & +F_{ba}P_{k-1|k-1,ab}^{T}F_{ab}^{T}+F_{bb}P_{k-1|k-1,bb}F_{ab}^{T}+Q_{k-1,ba} \end{array}$$

$Q_{k-1,ba}$ is the cross-covariance matrix of the process noise.

### 3.3. Marginalized Gromov Flow Particle Filtering for TBD

During the particle-flow update, a representative measurement-related state hypothesis is needed to guide particle migration. A clustering approach can identify potential target state hypotheses. The clustering method is described as follows: for a certain proportion (empirically set to 2%) of particles $x_{k,w}=\left\{x_{w}^{i},i=1,2,\cdots ,N_{w}\right\}$ with the highest weights in the proposal distribution at time *k*, the particle $x_{k,l_{max}}$ with the highest weight among the unassigned particles $x_{k,l}$ is selected as the cluster center. Particles satisfying (32) relative to this center are assigned to the same cluster. If a particle does not belong to any cluster, it is designated as the center of a new cluster. This process continues until all particles are assigned to a cluster. The weights of particles within each cluster are summed, and the cluster with the highest total weight is selected as the most likely target-associated cluster. The particle ${\hat{x}}_{k,l_{max}}$ with the highest weight within that cluster is selected as a representative state hypothesis to guide the particle flow.

(32) $$\frac{1}{\Sigma_{r}^{2}}{\left(r\left(x_{k,l_{max}}\right)-r\left(x_{k,l}\right)\right)}^{2}+\frac{1}{\Sigma_{b}^{2}}{\left(b\left(x_{k,l_{max}}\right)-b\left(x_{k,l}\right)\right)}^{2}<1$$

**Figure 2:**
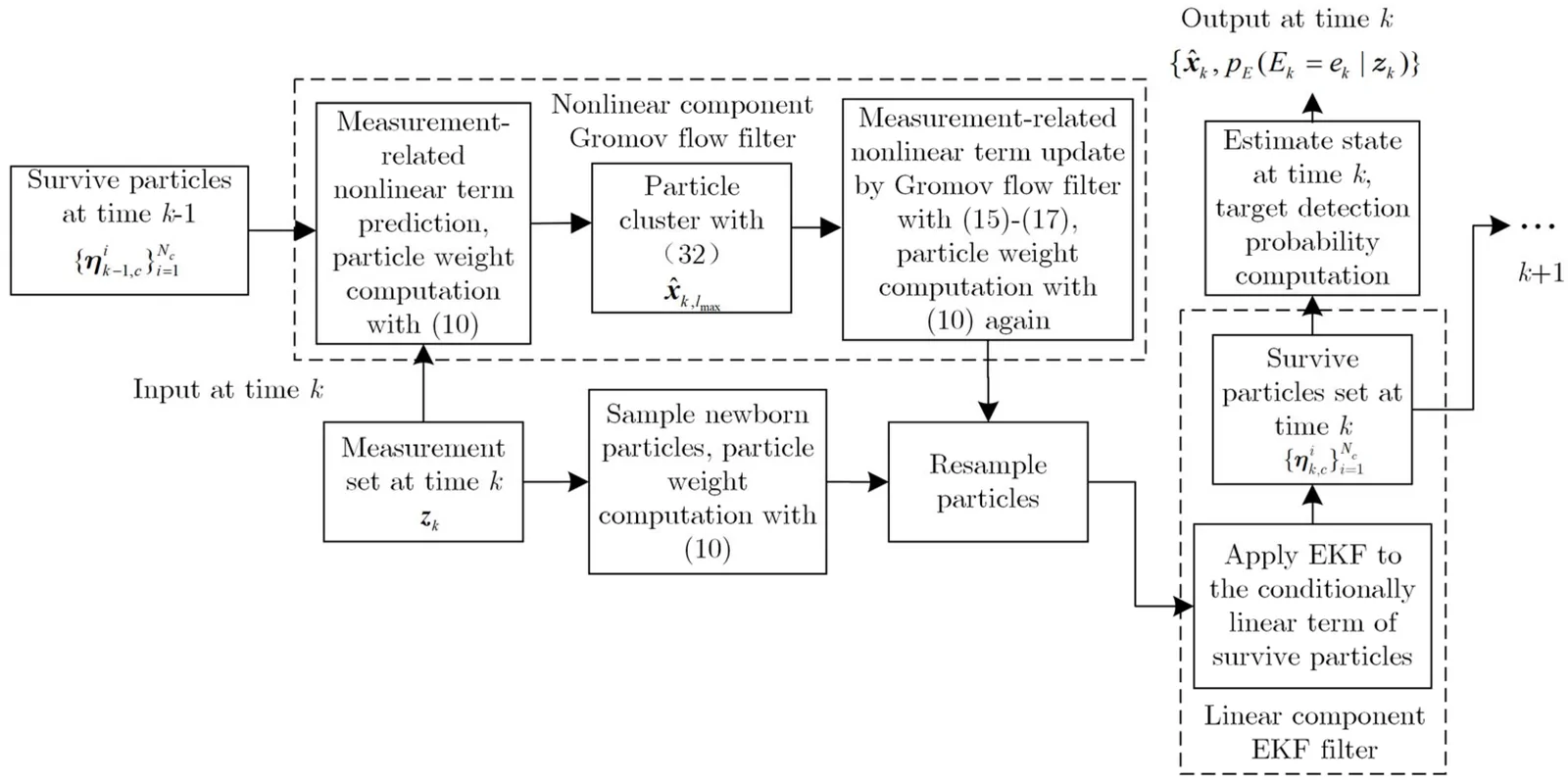
Block diagram of the MPFPF-TBD algorithm.

The MPFPF (Gromov) algorithm proposed in Section 3.2 is embedded into the PF-TBD framework [34] to obtain a marginalized particle flow particle filter-based track-before-detect (MPFPF-TBD) algorithm. Its implementation procedure is described below, the main notation used in the MPFPF-TBD algorithm is defined in Table 1, and the algorithm block diagram is shown in Figure 2.

Step 1: Sampling of newborn particles. For the measurement $z_{k}=\left\{z_{k}^{\left(i,j\right)},i=1,2,\cdots ,N_{r},j=1,2,\cdots ,N_{b}\right\}$ at time *k*, randomly draw a set of newborn particles ${\left\{\eta_{k,n}^{i}\right\}}_{i=1}^{N_{n}}$, and use (10) to compute the weights of newborn particles at time *k* ${\left\{\omega_{k,n}^{i}\right\}}_{i=1}^{N_{n}}\sim \frac{1}{N_{n}}l\left(z_{k}|\eta_{k,n},E_{k}\right)$.

Step 2: Particle-flow update of the measurement-related nonlinear state components. Apply the Gromov flow particle filter algorithm to predict and update the surviving particles ${\left\{\eta_{k-1,c}^{i}\right\}}_{i=1}^{N_{c}}$, obtaining the nonlinear measurement-related part of surviving particles at time *k*, denoted as ${\left\{\eta_{k,c,a}^{i}\right\}}_{i=1}^{N_{c}}$. Then, use the measurement at time *k* to compute the unnormalized surviving particle weight set ${\left\{\omega_{k,c}^{i}\right\}}_{i=1}^{N_{c}}\sim \frac{1}{N_{c}}l\left(z_{k}|\eta_{k,c,a},E_{k}\right)$.

Step 3: Weight calculation and particle resampling. Calculate the surviving term parameter $M_{c}=\left(1-p_{d}\right)p\left(E_{k-1}=e_{k-1}|\;z_{k-1}\right)\overset{N_{c}}{\underset{i=1}{\sum }}\omega_{k,c}^{i}$, the newborn term parameter $M_{n}=p_{b}p\left(E_{k-1}={\bar{e}}_{k-1}|\;z_{k-1}\right)\overset{N_{n}}{\underset{i=1}{\sum }}\omega_{k,n}^{i}$, and the normalization parameter $M_{k,j}=\frac{M_{j}}{M_{n}+M_{c}},j\in \left\{n,c\right\}$. Merge the nonlinear measurement-related part of surviving and newborn particles to obtain the nonlinear measurement-related part ${\left\{\eta_{k,a}^{i},\omega_{k}^{i}\right\}}_{i=1}^{N_{p}}={\left\{\eta_{k,c,a}^{i},\omega_{k,c}^{i}\right\}}_{i=1}^{N_{c}}\cup {\left\{\eta_{k,n,a}^{i},\omega_{k,n}^{i}\right\}}_{i=1}^{N_{n}}$ of the total particle set, and compute the particle weight coefficients ${\hat{\omega }}_{k}^{i}=M_{k,j}\omega_{k}^{i},j\in \left\{n,c\right\},i=1,2,\cdots ,N_{p}$. Resample the particle set ${\left\{\eta_{k,a}^{i},{\hat{\omega }}_{k}^{i}\right\}}_{i=1}^{N_{p}}$ to obtain the surviving particles ${\left\{\eta_{k,c}^{i}\right\}}_{i=1}^{N_{c}}$ at time *k*.

Step 4: Conditional update of the linear state components. For the set of linear parts $\eta_{k,b}$ of the surviving particles, use (26) and (27) to predict the conditionally linear components $x_{k|k-1,b}$ and $P_{k|k-1,b}$, and apply (28)–(31) to update $x_{k|k,b}$ and $P_{k|k,b}$. Merge the estimation results to obtain the state estimate ${\hat{x}}_{k}=E\{x_{k|k,a};x_{k|k,b}\}$ at time *k*.

Step 5: Compute the target existence probability $p_{E}(E_{k}=e_{k}|z_{k})=\frac{M_{n}+M_{c}}{M_{n}+M_{c}+p_{d}p(E_{k-1}=e_{k-1}|z_{k-1})+\left(1-p_{b}\right)[1-p(E_{k-1}=e_{k-1}|z_{k-1})]}$ and output the result.

## 4. Simulation Experiment

### 4.1. Scenario Settings and Simulation Parameters

The proposed MPFPF-TBD algorithm is compared with the conventional PF-TBD method [34], the LEDH-based PFPF-TBD method [21], and the spring model firefly algorithm-based particle filter TBD (SFA-PF-TBD) method [6]. The simulation includes 40 radar frames. The target appearance time is frame 7, and the disappearance time is frame 35, resulting in a presence duration of 28 frames. The radar sensor is located at the origin, with a scanning range $R_{T}\times \theta_{T}=\left[200,\;400\;m\right]\times [0,200\;m]$. In the simulation, the target moves on a two-dimensional plane, and its state is modeled using a CA model $x_{k}={\left[x_{k},y_{k},{\dot{x}}_{k},{\ddot{x}}_{k},{\dot{y}}_{k},{\ddot{y}}_{k}\right]}^{T}\in {\mathbb{R}}^{6}$. The state transition matrix $F=\left[\left[F_{aa},F_{ab}];[F_{ba},F_{bb}\right]\right]$ is specified by the blocks in (24). The states for newborn particles are configured as follows: position range $R_{p}\sim \left[250,\;350\;m]\times [50,\;150\;m\right]$, velocity range $V_{p}\sim \left[-1\;m/s,1\;m/s]\times [-1\;m/s,1\;m/s\right]$, and acceleration range $A_{p}\sim \left[-0.1\;m/s^{2},0.1\;m/s^{2}]\times [-0.1\;m/s^{2},0.1\;m/s^{2}\right]$. For the PFPF(LEDH)-TBD method and the MPFPF(Gromov)-TBD method, the particle flow parameters are configured as follows: number of iterations $N_{I}$ = 20, λ is selected using the exponential method, $\lambda =10^{-4+\frac{4n}{N_{I}}},\;n=0,1,\cdots ,N_{I}$. For the SFA-PF-TBD method, the parameters are set as follows: the initial attractiveness $\beta_{0}=1$, the absorption coefficient γ=0.01, the randomization parameter $\alpha_{0}=0.5$, the judgment radius $R_{j}=10$, the maximum particle proportion $P_{r\max}=0.5$, the spring activation threshold $P_{rth}=0.6$, the spring elastic coefficient $k_{s}=0.5$, and the maximum number of optimization iterations $N_{t\max}=20$. The experimental platform uses an Intel(R) Core(TM) i7-10875H CPU @2.3 GHz, equipped with 16 GB RAM, and runs on Windows 11 with MATLAB 2024b 64-bit. Other simulation parameters are shown in Table 2.

### 4.2. Comparison of TBD Performance Under Different SCR

This section analyzes the target tracking performance of different TBD methods under several low-SCR environments. Experimental settings are the same as those in Section 4.1 except for SCR.

**Figure 3:**
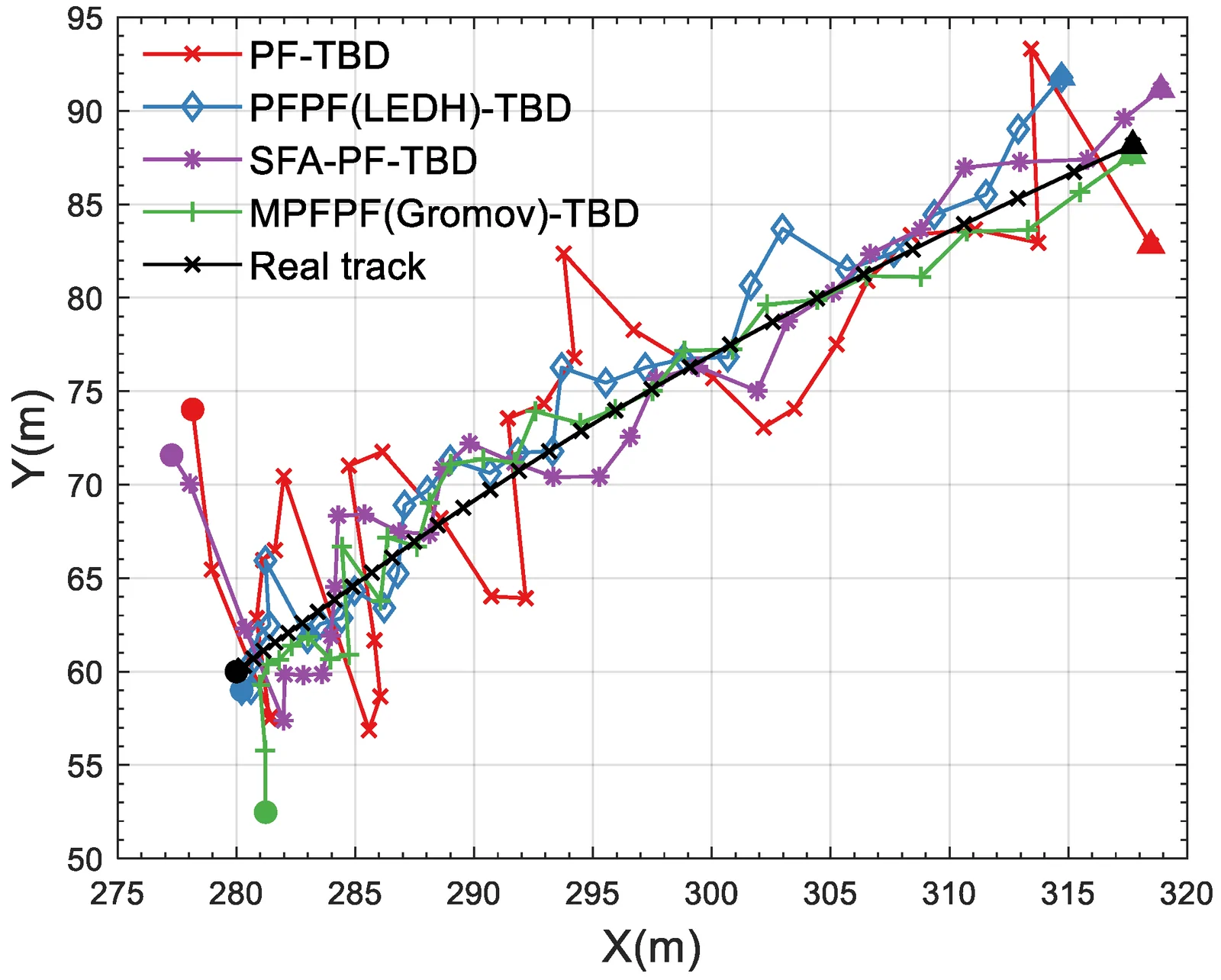
Trajectories of MPFPF-TBD, SFA-PF-TBD, PFPF-TBD and PF-TBD at the SCR of 6 dB.

Figure 3 compares the trajectories obtained by the four TBD algorithms at the SCR of 6 dB, where circles and triangles indicate the starting and ending points, respectively. The trajectories exhibit initial fluctuations because velocity and acceleration are not known when the target first appears. All four methods broadly follow the true trajectory, whereas PF-TBD shows larger deviations and MPFPF-TBD produces a more stable track after the initial stage.

**Figure 4:**
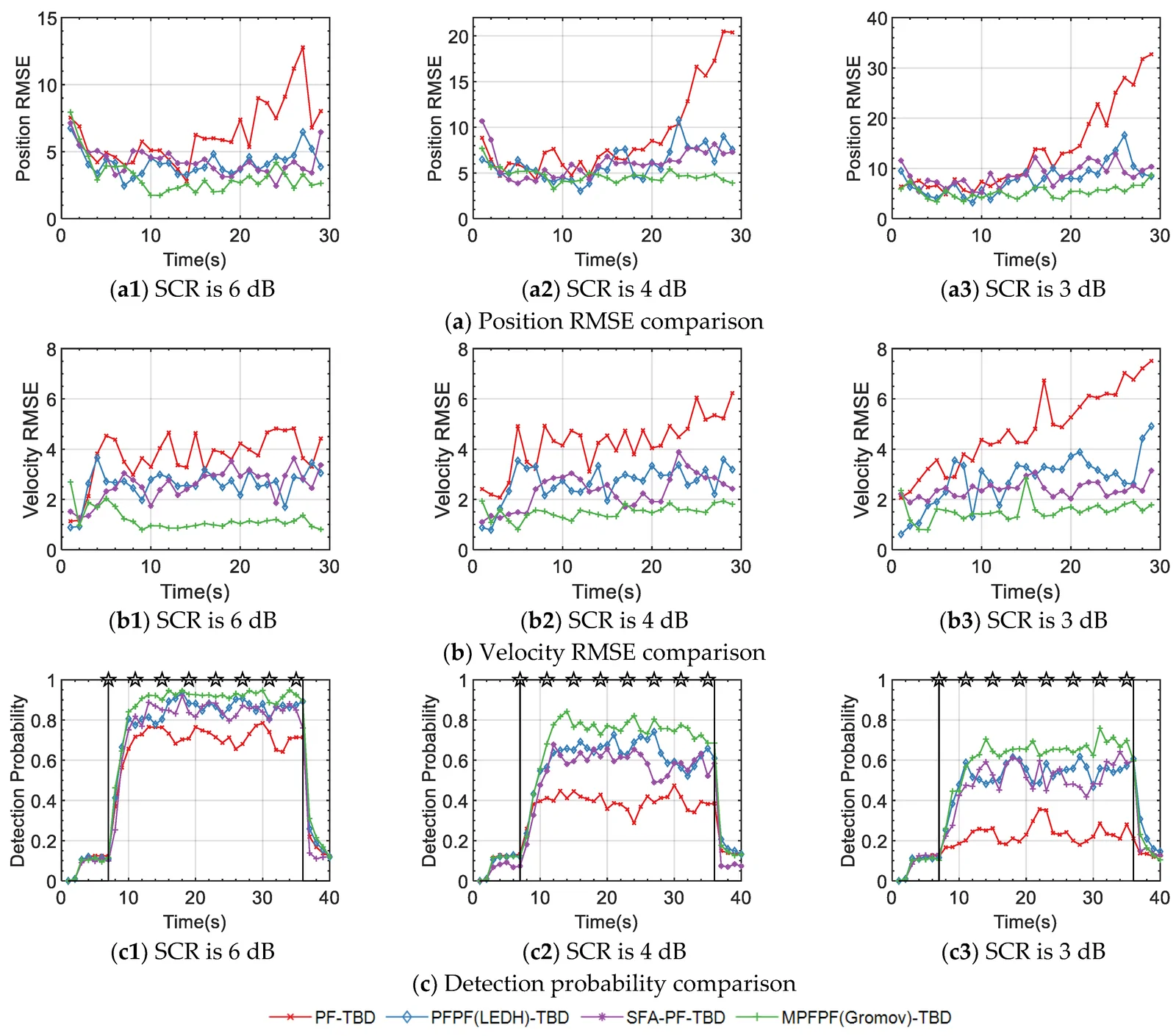
Comparison of TBD performance for MPFPF-TBD, SFA-PF-TBD, PFPF-TBD and PF-TBD under different SCR, the stars in Figure 4c indicate target exists.

Figure 4 compares the position RMSE, velocity RMSE, and target detection probability at SCRs of 6, 4, and 3 dB. Figure 4a presents the position RMSEs of the four algorithms under different SCR levels. The errors vary during initialization owing to random particle sampling. As the SCR decreases, the position RMSEs of PF-TBD and PFPF-TBD increase considerably in the later frames, particularly at SCRs of 4 dB and 3 dB. SFA-PF-TBD alleviates the error growth compared with PF-TBD, but its estimation error is generally higher than that of MPFPF-TBD. The proposed MPFPF-TBD maintains a relatively low and stable position RMSE after convergence. Figure 4b compares the velocity RMSEs. MPFPF-TBD generally achieves the lowest velocity estimation error under the three SCR conditions, while SFA-PF-TBD improves on PF-TBD and exhibits performance broadly comparable to PFPF-TBD. As shown in Figure 4c, MPFPF-TBD also provides the highest detection probability during the target-present interval. The detection probability of SFA-PF-TBD is higher than that of PF-TBD and is generally close to that of PFPF-TBD. These results indicate that MPFPF-TBD can improve detection and tracking robustness in low-SCR sea clutter.

### 4.3. Comparison of TBD Performance Under Different Dimensions

This section compares the target tracking performance of the four TBD methods under different dimensions. The simulation uses three state-space models of varying dimensions to form a set of comparative tests: for range-azimuth radar measurements, a constant-velocity (CV) model $x_{k}=\left[x_{k},{\dot{x}}_{k},y_{k},{\dot{y}}_{k}\right]\in {\mathbb{R}}^{4}$, a constant-turn (CT) model $x_{k}\in \;{\mathbb{R}}^{5}$, and a Jerk model $x_{k}\in {\mathbb{R}}^{8}$. Other experimental parameters remain the same as in Section 4.1.

**Figure 5:**
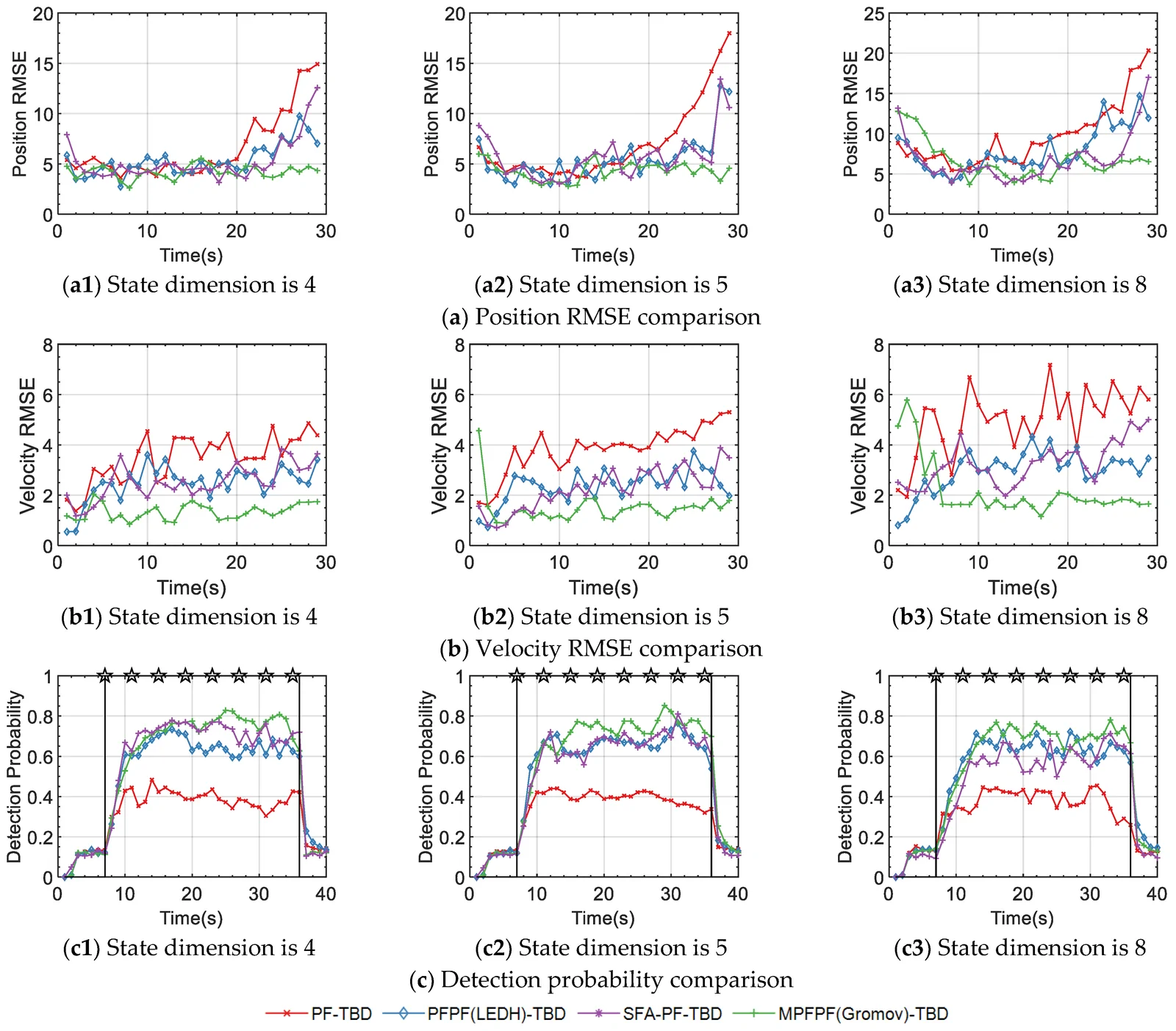
Comparison of TBD performance for MPFPF-TBD, SFA-PF-TBD, PFPF-TBD and PF-TBD under different dimensions, the stars in Figure 5c indicate target exists.

Figure 5a shows the position RMSEs of the four methods for different dimensional state models. The errors generally decrease during the initial convergence stage. In the later frames, however, PF-TBD exhibits pronounced error growth, and both PFPF-TBD and SFA-PF-TBD show varying degrees of degradation. By comparison, the position RMSE of MPFPF-TBD remains relatively low and stable across the three state-space dimensions. This result suggests that marginalization helps alleviate the deterioration of estimation accuracy associated with increasing state dimension.

Figure 5b compares the velocity RMSEs under the same state models. MPFPF-TBD generally achieves the lowest velocity RMSE and exhibits more stable estimation, especially in the higher-dimensional cases. SFA-PF-TBD reduces the velocity RMSE relative to PF-TBD in most frames but does not achieve the accuracy of MPFPF-TBD. Figure 5c presents the corresponding detection probabilities. After target appearance, the detection probabilities of the four methods increase and then fluctuate around relatively stable levels. MPFPF-TBD achieves the highest or near-highest detection probability across the tested dimensions, whereas PF-TBD shows the lowest detection performance. The detection probabilities vary less markedly with state dimension than the RMSE measures under the settings considered here.

### 4.4. Comparison of TBD Performance for MPFPF-TBD, SFA-PF-TBD, PFPF-TBD and PF-TBD Under Different Particle Numbers

This experiment investigates the tracking performance and computational costs of the four TBD methods. The number $N_{p}$ of particles varies between 500 and 3000, while other parameters are consistent with those in Section 4.1. The average execution time of the algorithms and the average position RMSE during the target presence interval are recorded to evaluate computational efficiency.

**Figure 6:**
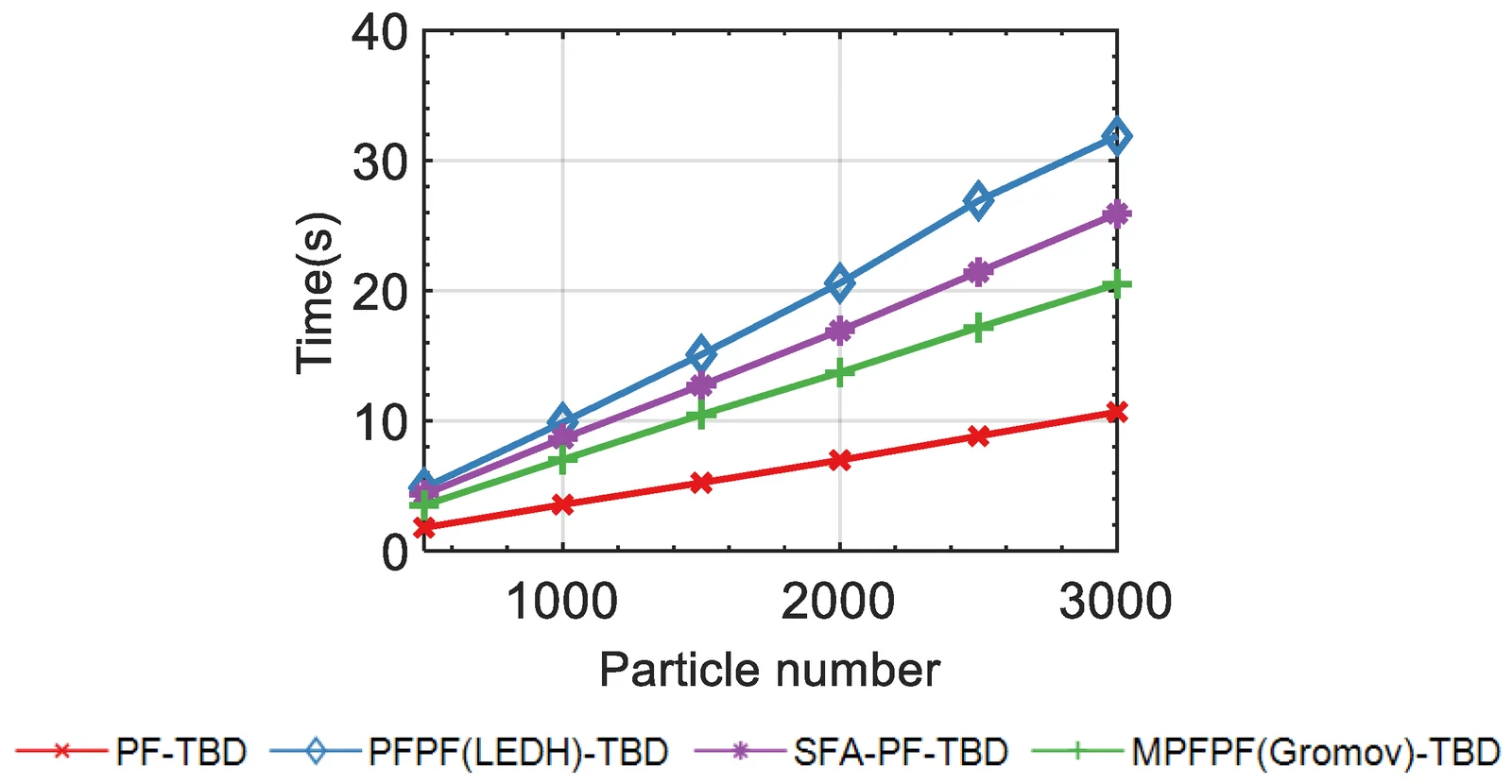
Runtime comparison of the four TBD algorithms under different particle numbers.

Figure 6 compares the average runtimes of the four algorithms as the particle number increases. The runtime rises approximately linearly with the number of particles for all methods. PF-TBD requires the least computation because it does not perform particle-flow or firefly-based particle optimization, while PFPF-TBD has the highest runtime. The runtime of SFA-PF-TBD lies between those of MPFPF-TBD and PFPF-TBD. Although MPFPF-TBD is more computationally demanding than PF-TBD, it is faster than both SFA-PF-TBD and PFPF-TBD. This advantage is consistent with the marginalization strategy, which restricts the Gromov flow computation to the measurement-related state components instead of the full target state.

Table 3 reports the average position RMSEs of the four methods at particle numbers of 1000, 2000, and 3000. At every particle number, MPFPF-TBD achieves the lowest RMSE, followed by SFA-PF-TBD, PFPF-TBD, and PF-TBD. The RMSE that MPFPF-TBD achieved with 1000 particles is lower than that of PF-TBD with 3000 particles and is close to that of PFPF-TBD with 2000 particles. Together with Figure 6, these results show that MPFPF-TBD provides a favorable balance between estimation accuracy and computational time among the four algorithms.

### 4.5. Comparison of TBD Performance Under Different K-Distribution Clutter Parameters

This experiment investigates the tracking performance of the four TBD methods under different clutter parameters. The shape and scale parameters used to generate sea clutter are varied, while the clutter parameters used in the likelihood models of all four algorithms remain fixed at α = 0.5 and β = 4. This setting also allows the robustness to clutter parameter mismatch to be evaluated. The average position RMSE, average velocity RMSE and the average detection probability of the algorithms during the target presence interval are recorded to evaluate detection and tracking.

The SCR is defined as $SCR=10\log_{10}\frac{{\bar{A}}^{2}}{2\alpha \beta }$, where $\bar{A}$ is the echo amplitude. In this experiment, the clutter shape parameter α and scale parameter β are varied jointly while maintaining αβ=2, and the SCR remains fixed at 4 dB. A smaller α corresponds to a heavier-tailed clutter amplitude distribution with more sea spikes, so the algorithms can be examined under different sea surface conditions with the same nominal SCR.

**Figure 7:**
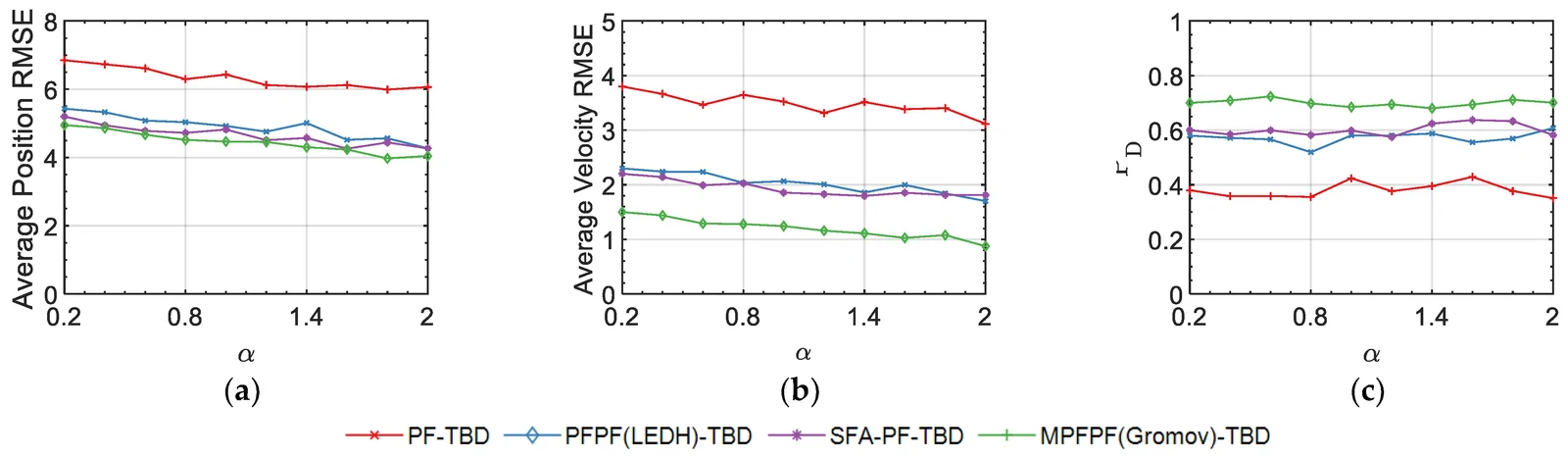
Comparison of TBD performance for MPFPF, SFA-PF, PFPF and PF under different shape parameters α and scale parameters β at an SCR of 4 dB, where αβ=2. (**a**) Average position RMSE comparison; (**b**) Average velocity RMSE comparison; (**c**) Average Detection probability comparison.

Figure 7a compares the average position RMSE as the clutter shape parameter increases from 0.2 to 2. The position errors of the four algorithms generally decrease as the clutter distribution becomes less heavy tailed. MPFPF-TBD maintains the lowest position RMSE across the tested parameter range, while SFA-PF-TBD and PFPF-TBD perform better than PF-TBD. Figure 7b shows the comparison for velocity estimation: MPFPF-TBD yields the smallest average velocity RMSE, followed by the two enhanced particle filtering baselines, whereas PF-TBD has the largest errors. In Figure 7c, MPFPF-TBD maintains the highest detection probability as the shape and scale parameters change. SFA-PF-TBD and PFPF-TBD provide intermediate detection performance, while PF-TBD remains the least effective. These results indicate that MPFPF-TBD is comparatively robust to variations in clutter impulsiveness and clutter parameter mismatch, including the more challenging heavy-tailed conditions at small shape parameter values.

### 4.6. Comparison Using Measured Sea Clutter with Simulated Target Echoes

**Figure 8:**
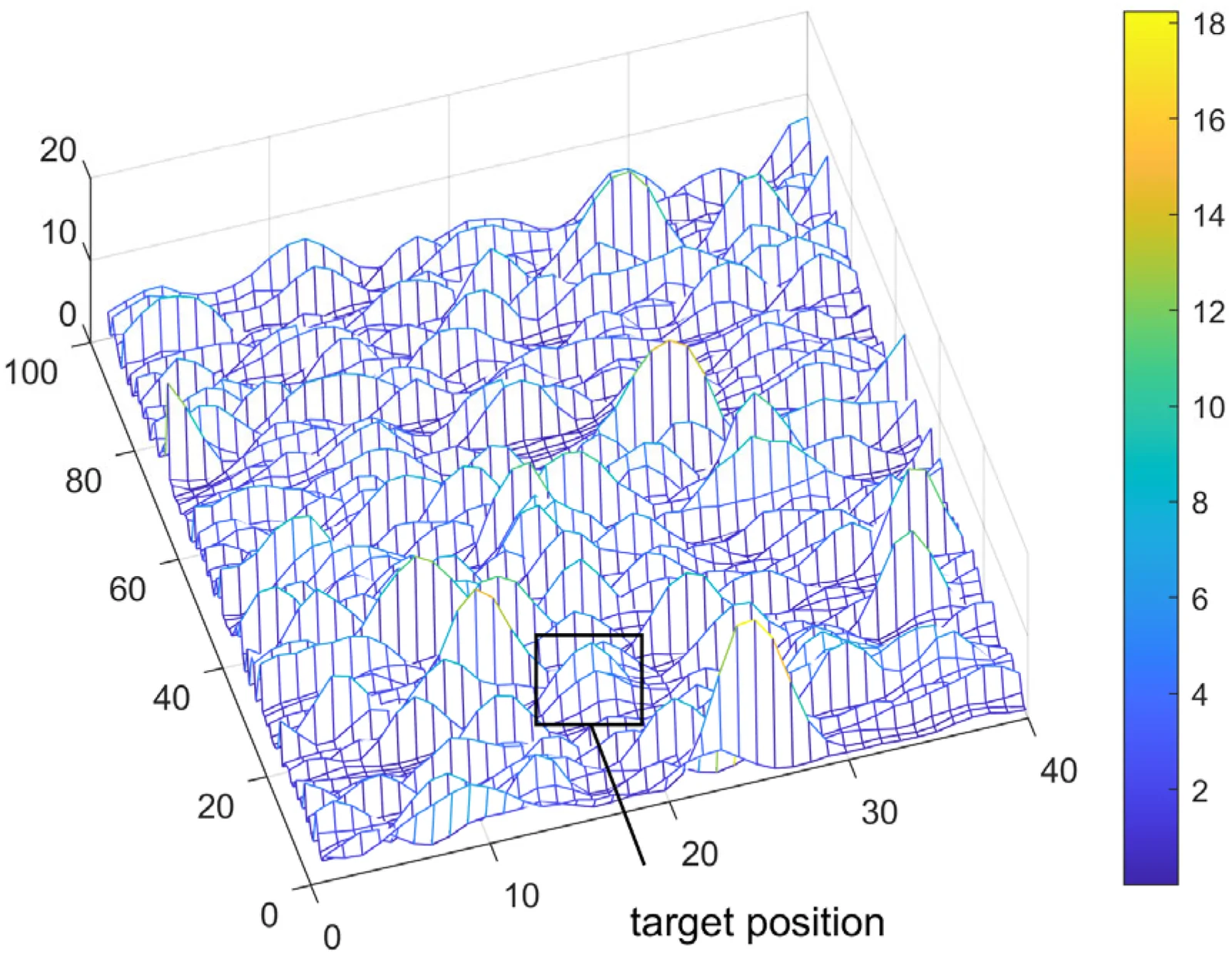
Side view of the sea clutter and weak target in the 13th frame.

**Figure 9:**
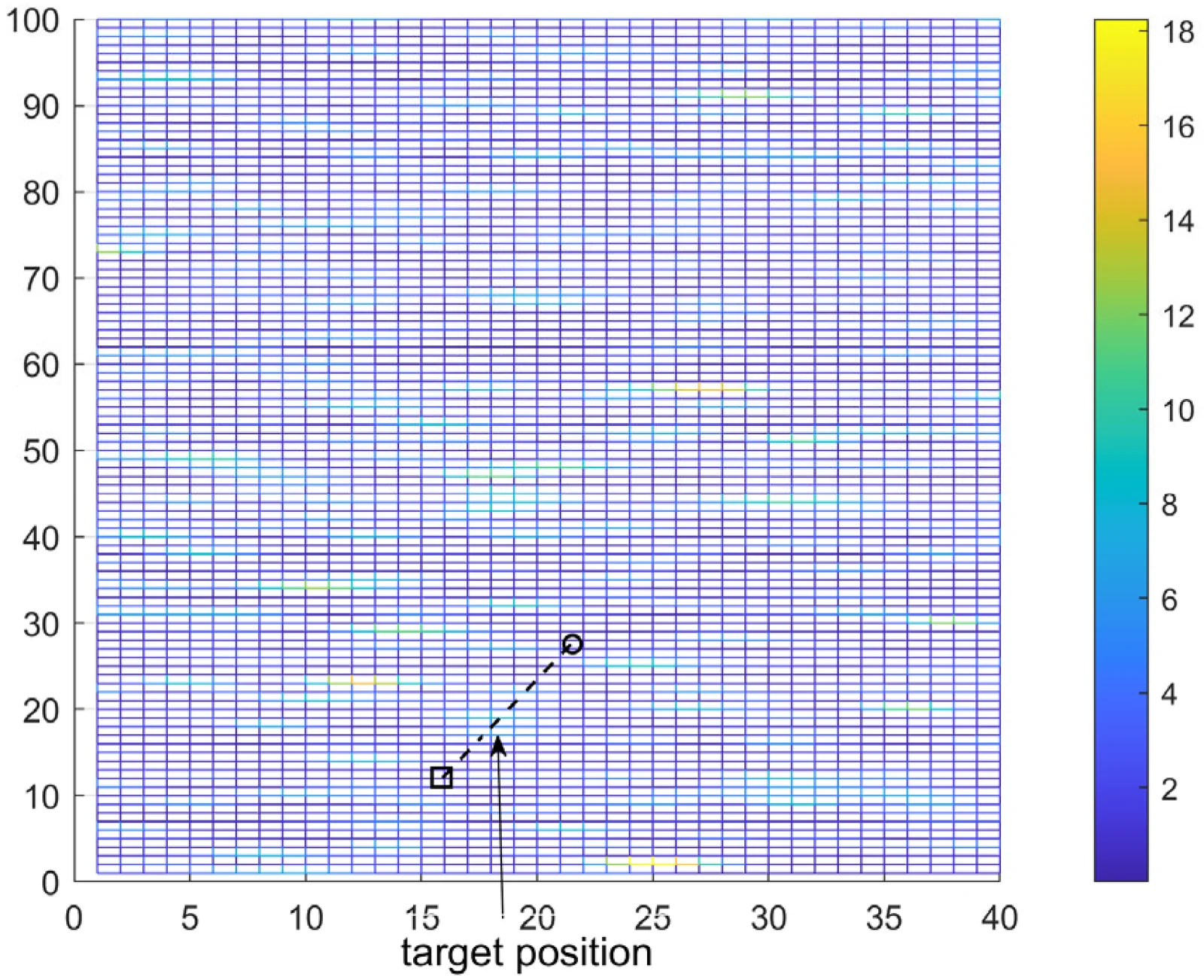
Top view of the sea clutter and weak target in the 13th frame, where square and circle mark the start and end points of the target.

Here, the dataset is constructed by superimposing a weak simulated target onto measured sea clutter data. The sea clutter dataset [35] was collected in 2020 at the First Bathing Beach in Yantai, using an X-band radar with a scan period of 2.5 s/r and a range resolution of 12 m. The total data length is 30 frames, the target SCR is 6 dB, and the target exists from frame 5 to frame 25. A snapshot of the measurement at frame 13 is shown in Figure 8 and Figure 9. It can be observed in Figure 8 that the background sea-spike clutter is highly spatially correlated, which is consistent with the physical nature of sea spikes. In Figure 9, square and circle mark the start and end points of the target, and arrows indicate the target position. The target energy is essentially submerged in the background clutter at this point, making visual discrimination difficult. In this challenging background, threshold-based detectors such as CFAR may produce numerous false alarms.

**Figure 10:**
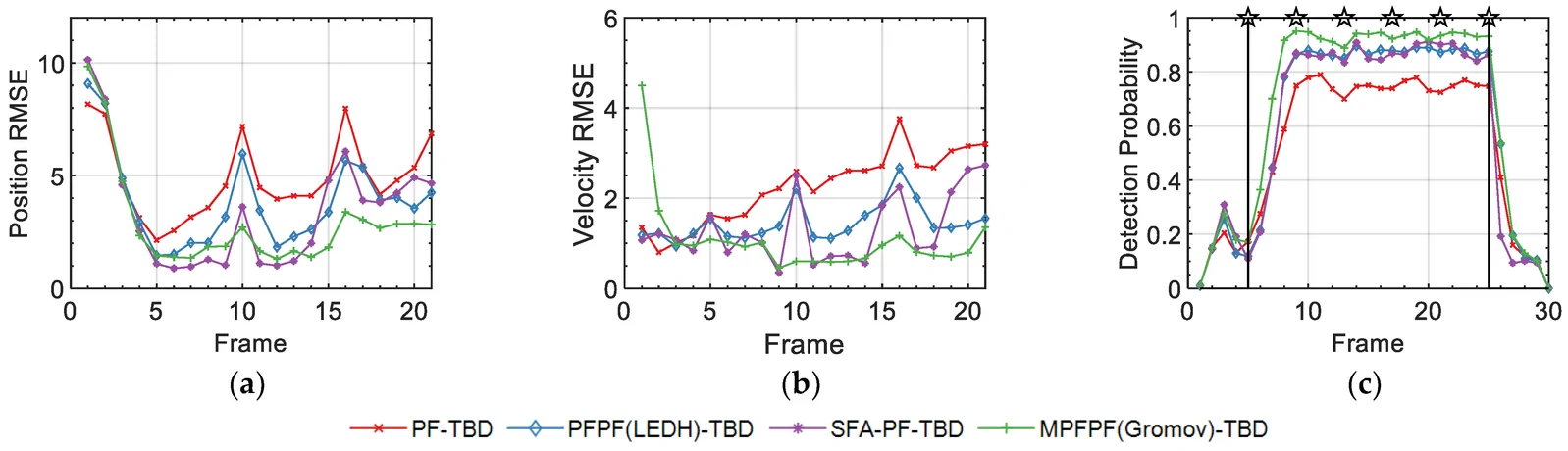
Comparison of position RMSE, velocity RMSE, and detection probability for the four TBD algorithms using measured sea clutter with simulated target echoes. (**a**) Position RMSE comparison; (**b**) Velocity RMSE comparison; (**c**) Detection probability comparison, the stars indicate target exists.

Figure 10 compares the four algorithms using measured sea clutter with synthetically injected weak target echoes. The position RMSE of the four methods decreases rapidly during the initial frames, but temporary increases appear around the 10th and 16th frames because of strong clutter near the target. MPFPF-TBD produces lower position RMSE than the other methods over most of the target-present interval, while both SFA-PF-TBD and PFPF-TBD generally improve on PF-TBD. Figure 10b shows that MPFPF-TBD maintains a relatively low velocity RMSE and is less affected by clutter-induced fluctuations. SFA-PF-TBD also improves velocity estimation relative to PF-TBD, although its error generally remains higher than that of MPFPF-TBD. In Figure 10c, MPFPF-TBD achieves the highest detection probability during target presence, SFA-PF-TBD and PFPF-TBD show intermediate performance, and PF-TBD yields the lowest probability. These results support the robustness of MPFPF-TBD in measured sea clutter. Nevertheless, the target echoes in this experiment are simulated and superimposed on measured clutter rather than obtained from actual marine targets. Such synthetic echoes may not fully reproduce real target characteristics, including radar cross-section fluctuations. Therefore, further evaluation using radar data containing actual marine targets is required.

## 5. Conclusions

To address the tracking problem of weak sea-surface targets in strongly cluttered environments, this paper proposed a marginalized Gromov flow particle filter-based track-before-detect algorithm for marine targets. The method is based on the Gromov flow particle filter and incorporates a marginalization strategy. The motivation is to use the Gromov flow to guide particle movement to improve the proposal distribution, while enhancing computational efficiency and alleviating the curse of dimensionality through marginalization. The results of Monte Carlo simulations and experiments using measured sea clutter data with simulated target echoes demonstrated that the proposed algorithm can effectively detect and track weak sea-surface targets in strong clutter. Compared with the conventional PF-TBD algorithm, LEDH-based PFPF-TBD and SFA-PF-TBD, the proposed method achieved favorable estimation and detection performance in the reported experiments and a lower runtime than the LEDH-based PFPF-TBD and SFA-PF-TBD baselines, although conventional PF-TBD remains faster.

## Figures and Tables

**Table 1:** Definitions of the main parameters and notation used in the MPFPF-TBD algorithm.

Symbol	Description
$N_{w}$	Number of high-weight particles selected for clustering
$N_{c}$	Number of surviving particles propagated from the previous time step
$N_{n}$	Number of newborn particles generated at the current time step
$N_{p}$	$\mathrm{Total}\;\mathrm{number}\;\mathrm{of}\;\mathrm{particles}\;\mathrm{in}\;\mathrm{the}\;\mathrm{combined}\;\mathrm{particle}\;\mathrm{set},\;\mathrm{where}\;N_{p}=N_{c}+N_{n}$
${\left\{\eta_{k,n}^{i},\omega_{k,n}^{i}\right\}}_{i=1}^{N_{n}}$	The newborn particles drawn at time *k*, where ${\left\{\omega_{k,n}^{i}\right\}}_{i=1}^{N_{n}}$ denotes weight calculated by (10)
${\left\{\eta_{k,c}^{i}\right\}}_{i=1}^{N_{c}}$	The surviving particles at time *k*
${\left\{\eta_{k,c,a}^{i},\omega_{k,c}^{i}\right\}}_{i=1}^{N_{c}}$	The nonlinear measurement-related state component of surviving particles at time *k*, calculated by particle flow, where ${\left\{\omega_{k,c}^{i}\right\}}_{i=1}^{N_{c}}$ denotes weight calculated by (10)
${\left\{\eta_{k,a}^{i},{\hat{\omega }}_{k}^{i}\right\}}_{i=1}^{N_{c}}$	The nonlinear measurement-related state component of particles at time *k*
$\eta_{k,b}$	Conditional linear state component at time *k*
$M_{c}$	Surviving term parameter
$M_{n}$	Newborn term parameter
$M_{k,j}$	Normalization parameter

**Table 2:** Simulation parameter settings.

Parameter	Value
Sampling time interval *T* (s)	1
Target birth probability $p_{b}$	0.1
Target death probability $p_{d}$	0.1
Radar range resolution ΔR (m)	0.5
Radar azimuth resolution Δθ (m)	0.5
Number of particles $N_{p}$	1000
Proportion of newborn particles	0.1
Proportion of surviving particles	0.9
Number of Monte Carlo simulations	100
Signal-to-Clutter Ratio (dB)	4
K-distribution clutter shape parameter α	0.5
K-distribution clutter scale parameter β	4

**Table 3:** Comparison of particle number and average position RMSE for the four algorithms.

Algorithm\Particle Number	1000	2000	3000
PF-TBD	6.87	6.00	5.57
PFPF-TBD	5.27	4.92	4.81
SFA-PF-TBD	5.20	4.82	4.49
MPFPF-TBD	4.96	4.43	4.05

## Data Availability

In this paper, we used the public data provided by Prof. N.B.L at https://radars.ac.cn/web/data/getData?dataType=DatasetofRadarDetectingSea, accessed on 1 January 2025.
